# Does context matter in misophonia? A multi-method experimental investigation

**DOI:** 10.3389/fnins.2022.880853

**Published:** 2023-01-04

**Authors:** Marta Siepsiak, Scott R. Vrana, Andrzej Rynkiewicz, M. Zachary Rosenthal, Wojciech Łukasz Dragan

**Affiliations:** ^1^Faculty of Psychology, University of Warsaw, Warsaw, Poland; ^2^Virginia Commonwealth University, Richmond, VA, United States; ^3^Department of Psychiatry and Behavioral Sciences, School of Medicine, Duke University, Durham, NC, United States; ^4^Institute of Psychology, Jagiellonian University, Kraków, Poland

**Keywords:** misophonia, decreased sound tolerance, psychophysiology, experiment, SCR, HR, context

## Abstract

**Introduction:**

Misophonia is a recently defined disorder in which certain aversive repetitive sounds and associated stimuli elicit distressing and impairing affective, behavioral, and physiological responses. The responses in misophonia may be stronger when the sound is produced by close friends and family, suggesting that the context in which a triggering cue occurs may have an important role in misophonia. As such, the goal of this study was to test experimentally whether the context of the sound source influences affective and psychophysiological responses to triggering stimuli in misophonia.

**Methods:**

Sixty one adults with misophonia and 45 controls listened to audio recordings (8 s) of human eating, animals eating, and human mouth smacking sounds (without eating). After a break, the same audio recordings were presented embedded within videos of human eating (congruent stimuli), animals eating (congruent stimuli), and, in the mouth smacking condition, with visually incongruent stimuli (hands playing in mud or in a bowl with a watery dough). Psychophysiological responses—skin conductance response (SCR) and heart rate (HR), and self-reported affective responses (valence, arousal, dominance) were gathered during the experiment in a laboratory.

**Results:**

Participants with misophonia assessed all the stimuli as more negative and arousing than the controls, and reported feeling less dominant with respect to the sounds. Animal and mouth smacking sounds were assessed by all the participants as less negative and arousing than human eating sounds, but only in the audio-video conditions. SCR data partially confirmed increased psychophysiological arousal in misophonia participants during an exposure to mouth sounds, but did not reflect the self-report changes in response to different contexts. Misophonia participants had deeper deceleration of HR than controls during human eating sound with congruent video stimuli, while there was no group difference during human mouth smacking with incongruent video stimuli.

**Conclusion:**

Results suggest that the context of mouth sounds influences affective experiences in adults with misophonia, but also in participants without misophonia. Presentation of animal eating sounds with congruent visual stimuli, or human mouth smacking sounds with incongruent stimuli, decreased self-report reaction to common misophonic triggers.

## Introduction

Misophonia is a newly defined disorder in which selective repetitive sounds or other associated stimuli elicit unpleasant affective, physiological, and behavioral responses that are accompanied by psychological distress and, over time, adversely impact one’s quality of life (Brout et al., 2018; Jager et al., 2020; Swedo et al., 2022). Misophonic responses are triggered usually, but not exclusively, by oral or nasal human-made sounds (Schröder et al., 2013; Enzler et al., 2021; Vitoratou et al., 2021; Swedo et al., 2022). Findings across studies indicate that the affective responses most commonly are irritation, anger, disgust, feeling trapped, anxiety, or rage (Schröder et al., 2013; Rouw and Erfanian, 2018; Jager et al., 2020).

Since misophonia was named and first described by Jastreboff and Jastreboff (2001), an unanswered empirical question is whether or to what extent misophonic responses are moderated by the context in which the sound is experienced, something that has been observed in clinical settings (Jastreboff and Jastreboff, 2014). Researchers have called for studies to be conducted that help elucidate a comprehensive understanding of the mechanisms underlying responses to misophonic stimuli, such as the context of triggering sounds (Brout et al., 2018). Additionally, the importance of context was identified in the recent and first consensus definition of misophonia (Swedo et al., 2022).

The context of the sound can be defined by actual environmental factors, such as sounds made by animals compared to humans, or sounds made by a close relative vs. a stranger.

For example, in Edelstein et al. (2013), participants with misophonia reported that their reaction to a trigger sound was stronger or limited to particular close friends or family members. Moreover, the majority of the participants in this study were not bothered by eating sounds produced by animals or babies. Jager et al. (2020) also reported that affective responses may not occur when a triggering sound is made by toddlers, adults with intellectual disabilities, or dementia sufferers.

However, the context of the sound can also be modified by the way one interprets or identifies the source of the sound, and this phenomenon has also been investigated in recent research studies. Edelstein et al. (2020) employed experimental manipulation of the sound source awareness. The authors reported that not only the actual context (i.e., assessment of human-made sounds as being more aversive than animal-made sounds), but also the perception of the source of the sound (human-made sounds assessed as being less aversive when identified as non-human made sound) can influence the misophonic reaction. These data seem to indicate preliminarily that both the actual eating sounds, as well as the belief about the source of the sound may influence the misophonic reaction. Several case studies also have highlighted the possible role of context in responses reported by patients with misophonia (Johnson et al., 2013; Alekri and Al Saif, 2019; Natalini et al., 2020; Cecilione et al., 2021).

One way the role of context has been clinically explored involves modification of a misophonic trigger for therapeutic purposes, wherein a study participant associated an eating sound with the sound of running in the snow to mitigate a misophonic reaction to this sound (Schröder et al., 2017). A similar manipulation was reported by Frank and McKay (2019), in which one of the participants was instructed to listen to the trigger sounds while imagining that similar sounds could be made by something different (e.g., a gorilla or a motor). The efficacy of these particular manipulations remains unknown (for example, modification of the sound’s context was one of many interventions that were used and it is not known which one was the most effective, and to what extent), however, they raise interesting hypotheses about the possible ways in which the role of context modified on a cognitive level may influence reactivity to misophonic sounds.

Most recently, several studies investigated the role of context and influence of cognitive processing of typical misophonic sounds on emotional reactions. Heller and Smith (2022) showed that misidentification of the sounds’ context (e.g., chewing food misidentified as stirring cereal) decreased their “aversiveness” rating among people with and without misophonia. Results pointing to the significance of the cognitive assessment of common trigger sounds were also found by Savard et al. (2022). In this study, the 20% with the most severe misophonia symptoms and the 20% with the least severe misophonia symptoms from a group of 300 individuals sampled from the general population were asked to assess and recognize sounds presented against multi-talker babble at various levels of signal-to-noise ratio. Both groups evaluated potential trigger sounds (orofacial) and unpleasant sounds (e.g., a child crying, dentist drill) as significantly more unpleasant than neutral sounds. Moreover, in the case of more favorable signal-to-noise ratios condition, when the sounds were more identifiable, they evoked more anger, disgust, and anxiety in all the participants. The difference in sounds’ rating was more pronounced in the highest misophonia symptoms group than in the lowest misophonia symptoms group, and in the case of the highest misophonia symptoms group the effect size was yet larger for trigger sounds than for unpleasant sounds.

Furthermore, Samermit et al. (2022) showed that the same potential trigger sounds are less unpleasant when paired with a video that is incongruent with the actual sound source, such as chewing sounds paired with a video of stepping on snow. Thus, the perception of the sound’s context was modified by experimentally manipulating the congruency between visual contextual cues and sounds triggers, impacting affective responses. In addition, a positive moderate correlation was found between the difference in the pleasure rating in these two conditions and misophonia symptoms.

Notably, the three latter studies examined adults from the general population, with low and high misophonia symptoms assessed using online questionnaires. As a result, it is possible that participants in these studies were not significantly impaired by misophonia symptoms in everyday life, or could have other sound intolerance conditions, such as hyperacusis or phonophobia. For example, in Savard et al. (2022) only 6 out of 66 participants from the group with high misophonia symptoms met the cut-off for misophonia on the MisoQuest (Siepsiak et al., 2020). Similarly, in Samermit et al. (2022), 14 out of 101 participants met the cut-off for moderate or higher impairment misophonia on the Misophonia Questionnaire (Wu et al., 2014). Therefore, the results should be replicated in people with misophonia symptoms significantly affecting their lives, ideally using clinical interviews as an assessment method in lieu of questionnaires.

Responses to trigger sounds in misophonia sufferers have also been studied using psychophysiological measures. Changes in heart rate (HR) and skin conductance response (SCR) are associated with autonomic nervous system response to affective stimuli (Levenson, 1992, 2014; Cacioppo et al., 2000). In the study by Edelstein et al. (2013), students with misophonia had greater mean SCR while listening to misophonic trigger sounds (chosen individually for each of the participants) than students without misophonia. In addition, Kumar et al. (2017) found that only human-made sounds, but not other aversive and neutral sounds, evoked SCR and HR increases and in misophonia sufferers more than in controls. Similarly, in a study by Schröder et al. (2019), misophonic sounds elicited higher HR than aversive and neutral sounds in the misophonia group. These results demonstrate that specific, repetitive sounds evoke autonomic responses in people with misophonia, consistent with their self-reports. They are also in line with findings of increased HR responses to extremely aversive stimuli.

Phasic HR to a discrete stimulus is usually characterized by an initial deceleration that indicates orienting and information intake, followed by HR acceleration responsive to arousal and action readiness (Bradley et al., 2001; Witvliet and Vrana, 2007). Negatively valent stimuli are particularly significant and often produce a larger orienting response than neutral stimuli (Bradley et al., 2001). Cardiac deceleration to negative visual stimuli is especially large and sustained without subsequent acceleration unless the stimulus is extremely aversive, such as a person with a severe phobia viewing a picture of a phobic object, or prolonged in duration. For example, Acute Stress Disorder and PTSD patients showed (Elsesser et al., 2004) acceleration of HR while viewing trauma-related pictures (notably, those with PTSD had slight initial HR deceleration), while deceleration of HR was observed in controls, whereas during exposure to aversive, but not trauma-related, pictures, HR in both groups decelerated. A slight deceleration followed by acceleration of HR in response to pictures related to injuries was also observed in war or torture survivors diagnosed with PTSD, whereas the healthy controls and trauma resilient survivors showed steep and deep HR deceleration, followed by slow return toward the baseline level (Adenauer et al., 2010). In a study by Rosenbaum et al. (2020), where the stimuli lasted longer, spider phobia patients had higher mean HR during a presentation of spider pictures than during pictures of domestic animals, while this change was not observed in controls. In a similar study (Wannemüller et al., 2015), participants with dental phobia had acceleration of HR while being exposed to pictures and noises related to their phobia, and deceleration of HR during exposure to neutral stimuli, whereas deceleration of HR during exposure to all the stimuli was observed in controls. SCR, like initial HR deceleration, is responsive to orienting and information intake, and is often observed in response to arousing stimuli, whether negative or positive (e.g., dangerous or threatening stimuli, but also erotic, sport-related, or funny stimuli; Bradley et al., 2001; Bos et al., 2013; Nigbur and Ullsperger, 2020).

The primary goal of the present study was to investigate whether the context, either set by environmental factors (human vs. animal-made sounds) or by manipulation of the sound’s source (congruent vs. incongruent visual stimuli) influences self-report and psychophysiological responses to common misophonic stimuli in a misophonia and a control group. Mouth sounds were presented either as an auditory cue alone or, in audio-video condition, with a congruent video (human or animal eating sounds) or with an incongruent video (human mouth smacking sounds presented against videos of human hands).

The misophonic response was assessed via self-report on the three primary dimensions of emotional evaluation (Mehrabian and Russell, 1974): valence (pleasure-displeasure), arousal (arousal-relaxation), and control (dominance-submission). Physiological reaction was assessed with phasic HR and SCR.

It was hypothesized that:

(1) Compared to a healthy control group of adults, the misophonia group would assess all stimuli as more negative, more arousing, and as feeling less dominant toward them than the controls, regardless of context;

(2) Higher SCR and less pronounced deceleration of HR would be observed in people with misophonia in response to all stimuli, in comparison to the control group;

(3) In the audio-video condition (but not in the audio condition) the misophonia group would assess animal sounds (congruent) and human mouth smacking sounds (incongruent) as less negative, less arousing, and as feeling more dominant toward them than toward humans eating sounds (congruent), whereas this effect would not be observed in the control group;

(4) In the audio-video condition (but not in the audio condition), the misophonia group would have reduced HR response (deeper or more sustained deceleration) and SCR (i.e., SCR will be lower) in response to animal (congruent), and human mouth smacking sounds (incongruent) than in response to the human eating sounds (congruent), whereas this effect would not be observed in controls;

(5) Presenting the sounds with videos will decrease the rating of negative valence, decrease arousal, and increase the dominance in the misophonia group in response to animal-made sounds (congruent) and human mouth smacking sounds (incongruent), but not to human eating sounds (congruent). This effect will not be observed in the control group;

(6) Presenting the sounds with videos will reduce HR reaction (deeper or more sustained deceleration) and SCR responses (SCR will be lower) in comparison to the audio condition in the misophonia group in response to animal-made sounds (congruent) and human mouth smacking sounds (incongruent), but not to human eating sounds, whereas this effect will not be observed in controls.

## Materials and methods

The Ethics Committee at the Faculty of Psychology, University of Warsaw (no. 29/05/2018) approved this study. This study was a part of a larger parent misophonia project conducted at this university.

### Participants

The study was advertised in social media, radio, local and online news (the language included: *Do certain sounds drive you mad? Can you not stand some particular sounds? Or maybe you do not have any sound over-responsivities?*). Individuals willing to take part in the study completed the online recruitment questionnaire, indicated whether they had any sound sensitivities, completed a questionnaire to assess misophonia (MisoQuest; Siepsiak et al., 2020), and provided demographic and contact information for study scheduling. A total of 131 people participated in the experiment, and the data of 106 participants who met the criteria for the group inclusion were analyzed: 61 participants with misophonia and 45 healthy controls without any sound over-responsivity took part in the study. Individuals with heart disease, substance addiction, or facial hair (as we collected facial EMG data for another study, not described here) were excluded from the study. Participants were asked to avoid caffeine or energy drinks 3 h before the experiment. They signed an electronic version of consent and were remunerated with 50 PLN (12.5 USD).

Because the age distribution in both groups was right-skewed, in order to compare whether there were age differences between the groups, a U Mann-Whitney test was conducted. There was a significant age difference between misophonia (*Mdn*^1^ = *30;* range: 19–55) and controls (*Mdn* = *23*; range: 19–45), *U* = 757.50, *z* = −3.468, *p* < 0.001. In order to compare the gender ratio between the groups, a Chi-Square test was conducted. There were significantly [(*x*^2^ = 1; *N* = 105) = 3.95; *p* = 0.047] more females in the misophonia group (90%) than in the control group (76%).

### Misophonia assessment and the control group assignment

Each of the invited participants was assessed by psychologists trained in assessment of misophonia to conduct face-to-face interviews. Misophonia assessment for group inclusion was based on criteria proposed by Schröder et al. (2013). Specific eligibility criteria included: (a) experiencing immediate psychophysiological reaction in response to human produced oral or nasal sounds, (b) recognizing anger as a dominant (but not necessarily sole) emotion evoked by these sounds, and not fear or anxiety, (c) perceiving these emotions as excessive and overwhelming (d) avoiding exposure to these sounds, and in case of being exposed—reporting a significant distress caused by these sounds, (e) reporting a significant decrease in quality of life due to this sound over-responsivity. Eligibility for the control group was to report not having any sound over-responsivity. Participants who during the interview reported being occasionally bothered by sounds that are commonly perceived as unpleasant, (e.g., styrofoam sounds or sounds of sliding a fork over a plate) were included in the control group. Furthermore, participants who reported that they disliked eating sounds but never believed it was a problem for them were included in the control group. Participants with a variety of auditory over-responsivities (25 individuals) significantly affecting their lives who did not meet the misophonia criteria were not considered misophonia participants, so their data were not analyzed (e.g., participants with presumed hyperacusis or those whose main triggers were neighbor sounds, snoring, siren or barking sounds, or those whose main emotion when exposed to their trigger was fear or anxiety, not anger or extreme irritation).

Additionally, the validity of group inclusion was confirmed with a questionnaire for assessing misophonia—MisoQuest, administered online at the time of participants’ recruitment, a 14-item questionnaire with good reliability (Cronbach’s alpha = 0.95) and stability (intraclass correlation coefficient = 0.84; Siepsiak et al., 2020). The results of Welch’s *t*-test indicated a significant difference [*t*(53.122) = 13.554; *p* < 0.001; Cohen’s *d* = 2.81] in the severity of misophonia symptoms between misophonia (*n* = 61; *M* = 64.57; *SD* = 4.9; range: 44—70) and controls (*n* = 45; *M* = 36.71; *SD* = 13.13; range: 14—59). Because the data from MisoQuest were not normally distributed according to Shapiro-Wilk Test [*W*(59) = 0.832, *p* < 0.001] but there was a normal distribution in the control group [*W*(43) = 0.960, *p* = 0.136], and the number of observations in each group was > 20, it was decided to use a parametric test, with a correction for unequal variances (Schmider et al., 2010; Blanca et al., 2017; George and Mallery, 2019).

### Behavioral measurement

**FIGURE 1:**
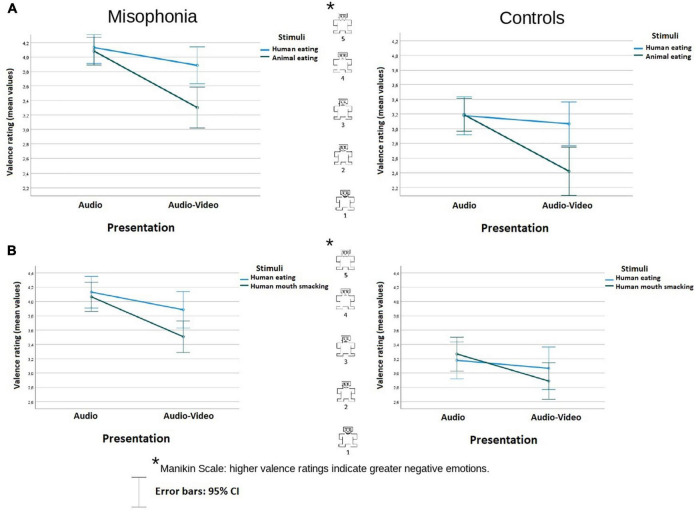
Valence rating (mean values) of the stimuli in audio and audio-video conditions in misophonia and the control group, separately for the analysis **(A)** and the analysis **(B)**. Higher valence ratings indicate greater negative emotions. The distances between the scale values were identical (it is a linear scale).

**FIGURE 2:**
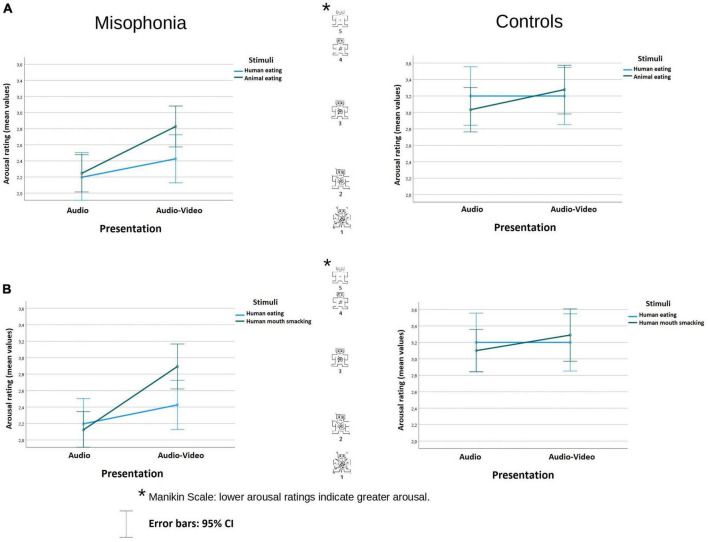
Arousal rating (mean values) of the stimuli in audio and audio-video conditions in misophonia and the control group, separately for the analysis **(A)** and the analysis **(B)**. Lower arousal ratings indicate greater arousal. The distances between the scale values were identical (it is a linear scale).

**FIGURE 3:**
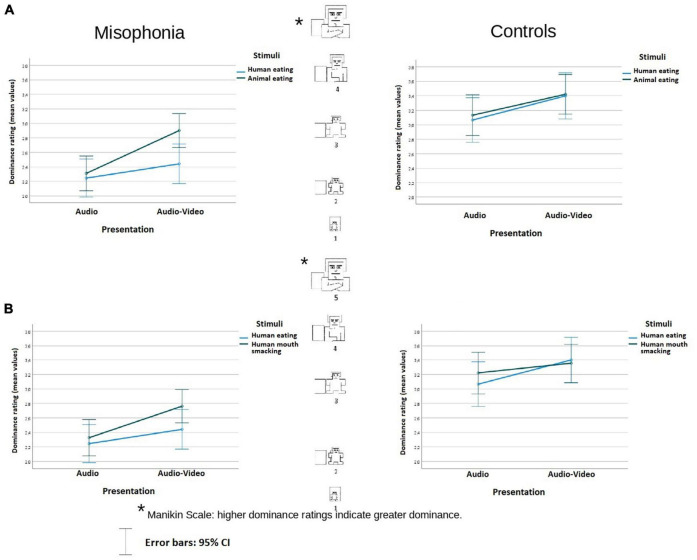
Dominance rating (mean values) of the stimuli in audio and audio-video conditions in misophonia and the control group, separately for the analysis **(A)** and the analysis **(B)**. Higher dominance ratings indicate greater dominance. The distances between the scale values were identical (it is a linear scale).

Self-reported affective responses were assessed with the Self-Assessment Manikin scales (Bradley and Lang, 1994). These are pictorial scales for assessing affective response to stimuli. It allows for measurement of three dimensions of emotions– valence, arousal, and dominance–each on a 1–5 scale. Each scale is depicted in Figures 1–3. The instruction (Imbir, 2016, p. 3) that was used in our study for the valence rating, was as follows: “The first picture shows a person who is obviously elated—relevant experiences could include fun, delight, happiness, relaxation, satisfaction, or repose. The last picture shows a person who is clearly distressed—relevant experiences could include panic, irritation, disgust, despair, defeat, or crisis. The remaining pictures depict intermediate states.”

For the dominance (Imbir, 2016, p. 3): “The first picture shows an individual who feels a lack of control and agency—relevant states could include subordination, intimidation, subjugation, withdrawal, submission, or resignation. The last picture shows a person who is dominant and in control of the situation—relevant states include control, influence, being important, dominant, recognized, or decisive.” For arousal (Imbir, 2016, p. 3): “The fir picture shows an individual who is very calm, almost sleeping—relevant states could include relaxation, tranquility, idleness, meditation, boredom, or laziness. The last picture shows an individual who is bursting in arousal—relevant states could include excitation, euphoria, excitement, rage, agitation, or anger.”

### Psychophysiological measurements

Galvanic skin response (GSR) and electrocardiography (ECG) were recorded with the BIOPAC MP-150 system through AcqKnowledge software. For GSR measurement, the EDA100C amplifier was used. The Ag-AgCl electrodes filled with dedicated gel were placed on the distal phalanges of the index and middle finger of the non-dominant hand. SCR level was measured with 5 mikroS/V gain and recorded at the rate of 2,000 samples per second. We decided to use as weak hardware filters as possible (no high-pass and 10 Hz low-pass) and then after visual inspection we noticed that offline software filters were not necessary. The SCL data were visually inspected for artifacts in AcqKnowledge, and then preprocessed in Matlab. Further statistical analyses were made in IBM SPSS Statistics 28. The period of 1-s before the onset of the main stimuli served as a baseline and was subtracted from eight 1-s periods after the onset of the stimuli—thus the SCR was obtained. Therefore, the negative values in SCR indicate the decrease in skin conductance level (SCL) in relation to the baseline.

For ECG, we used the ECG100C and a 3-lead arrangement of electrodes, which provides a clear shape of the ECG waveform and does not require removing the upper part of clothing. Two active electrodes were attached on the sides of the chest, and an inactive electrode was attached at the lower part of the sternum. Self-adhesive Ag/AgCl electrodes and standard ECG gel were used. Similar to SCL measurement, we limited hardware filters to minimum (150 Hz low-pass and 0.05 Hz high-pass). Each of the HR data recordings was visually inspected for artifacts in Acqknowledge, followed by preprocessing in the Matlab environment—no additional software filters were necessary to identify R-waves correctly. All statistical analyses were made in IBM SPSS Statistics 28. In order to check whether heart rate phasic response to stimuli differs between the groups, the average HR was calculated separately for 8 post-trigger 1-s periods using the standard method to derive HR from a measurement lasting less than a minute (Berntson et al., 2016). HR during the 1-s before the trigger was then subtracted from HR of each second after the trigger onset in order to create change scores.

### Stimuli and apparatus

Five audio recordings and five audio-video recordings with the same sounds—three movies from YouTube (ASMR Suna, 2018; Mayapolarbear, 2019; SAS-ASMR, 2019) and two recorded by the first author of the study served as stimuli: human eating, animal eating, and human mouth smacking sounds, without involving food inside (these stimuli aimed to be equivalent to human eating sounds- not having food inside the mouth while recording the audio sounds was unintentional). In the first condition only the audio cues were used. In the second condition, the sounds were presented either with a congruent video (animal eating videos and human eating video) or with incongruent videos of hands playing in mud or in watery dough, synchronized with the sounds. The incongruent video aimed to modify the context of the sound. Initially, 6 stimuli were planned, but due to technical issues, one of the two human eating stimuli was presented to fewer than half of the participants and was not analyzed. Therefore, in further analysis, average values from 2 animal-eating stimuli and 2 human mouth smacking stimuli rating and responses were analyzed. The procedure was displayed in PsychoPy (Peirce et al., 2019) and programmed in Python language (Van Rossum and Drake, 1995). The markers were sent to Acqknowledge software through a parallel port.

### Procedure

The participants sat on a chair in front of a computer with speakers and a keyboard in an air-conditioned room. During the experiment, they were alone. Before the experiment started, the research assistants placed the electrodes and explained the procedure. The participants were told that sounds or videos with sounds would be presented to them. Participants were informed that the sounds and videos could be neutral, aversive, or pleasant, depending on the individual’s preferences, and that they could press a security button or switch off the sound to stop the experiment immediately. They were asked to assess their feelings in response to the sounds and videos on the pictorial scales (see “Behavioral measurement” section). The answers were given after each single stimulus, by typing numbers, from 1 to 5, on the computer keyboard. The description of the pictorial scales was also displayed on the computer screen at the beginning of the experiment. The stimuli were displayed after the answers were given, so there was no time limit to give an answer.

Before the experiment began, there was a 5-min resting baseline period. The participants were asked to relax in the chair in front of a blank screen, and the level of psychophysiological signals was recorded and sent to Acqknowledge software through Biopac System. The sounds were presented under speakers, at the volum similar to eating sounds in real life, the same for each participant. During the first part of the experiment (A), participants listened to the audio recordings (animal eating sounds, human eating sound, and human mouth smacking sounds). They did not receive any information from the experimenter regarding the source of the sounds. In the second part (B), after a break for other tasks (a questionnaire for assessing temperamental traits and another experiment with audio-video that are not described in this paper), the participants were presented with the same stimuli, but this time the audio recordings were accompanied by videos (congruent animals eating videos, congruent human eating video, and incongruent to human mouth smacking sounds—video of hands). Each of the stimuli (of 8-s duration) was presented once, in a randomized order. Between each stimulus, there was an interstimulus interval—a black fixation cross displayed in the center of the white screen, with a duration of 8, 10, and 12 s, selected randomly.

## Results

### Behavioral data

The data were analyzed in IBM SPSS Statistics 28. Visual inspection of box plots revealed one outlier (in the control group valence assessments of the animal-eating sound in the audio-visual condition), which did not impact the results, so was not removed. Levene’s test was non-significant in all cases, except for the Arousal assessment in human eating sounds in the audio-video condition (*p* = 0.007; Equality of Covariance Matrices *p* = 0.002). In order to explore whether the type of visual information about source of the sounds has an influence on the emotional reaction, separate mixed ANOVAs^2^ were conducted on the participants’ ratings of valence, arousal, and dominance, with (a) Group (misophonia, control), Stimuli (human, animal), and Presentation (audio, audio-video) as variables, with the latter two being repeated measures to test the hypothesis of the effect of adding congruent visual information on the actual sound’s sources and (b) Group (misophonia, control), Stimuli (human eating, human mouth smacking sounds), and Presentation (audio, audio-video) to test the hypothesis of the effect of presenting an actual human mouth smacking sounds with incongruent video.

When the sphericity assumption was not met, Greenhouse-Geisser corrected degrees of freedom and epsilon values were reported. Bonferroni *post-hoc* tests with correction for multiple comparisons were conducted.

#### Valence

In the analysis involved human eating congruent stimuli and animal eating congruent stimuli, participants with misophonia reported the sounds (an average across conditions) overall as more negative (*M* = 3.85, *SE* = 0.09) than did controls (*M* = 3.00, *SE* = 0.11), Group *F*(1, 104) = 38.41, *p* < 0.001; η^2^*_*p*_* = 0.27. There was no interaction between the Group and other variables.^3^ There was an interaction of Stimuli × Presentation *F*(1, 104) = 17,95, *p* < 0.001; η^2^*_*p*_* = 0.15. Pairwise comparison showed that while there was no difference between the stimuli in the audio condition (*p* = 0.777), in audio-video condition, and human eating (*M* = 3.64, *SE* = 0.074) was assessed as significantly more negative than animal eating (*M* = 2.86, *SE* = 0.11), *p* < 0.001. Moreover, while there was no difference in the human eating rating between audio and audio-video conditions (*M* = 3.65, *SE* = 0.09 vs. *M* = 3.48, *SE* = 0.1, *p* = 0.088), animal-made sounds were assessed as more positive in the audio-video condition (*M* = 2.86, *SE* = 0.11, *p* < 0.001) than in the audio condition (*M* = 3.64, *SE* = 0.07), *p* < 0.001. The data are illustrated in Figure 1A.

In the analysis of human eating congruent stimuli and human mouth smacking incongruent stimuli, participants with misophonia also reported the sounds overall as more negative (*M* = 3.9, *SE* = 0.09) than did controls (*M* = 3.1, *SE* = 0.10), Group *F*(1, 104) = 35.18, *p* < 0.001; η^2^*_*p*_* = 0.25. There was no interaction between the Group status and other variables. There was an interaction of Stimuli × Presentation *F*(1, 104) = 5,831, *p* = 0.017; η^2^*_*p*_* = 0.053. Pairwise comparison showed that while there was no difference between the stimuli in the audio condition (*p* = 0.87), in the audio-video condition human eating congruent stimuli (*M* = 3.67, *SE* = 0.08) were assessed as significantly more aversive than human mouth smacking incongruent stimuli (*M* = 3.2, SE = 0.09), *p* = 0.005. Moreover, while there was no difference in the human eating rating between audio and audio-video conditions (*p* = 0.09), human mouth smacking sounds were assessed as less negative in the incongruent audio-video condition (*M* = 3.2, *SE* = 0.09), compared to the audio condition (*M* = 3.67, *SE* = 0.08), *p* < 0.001. The data are illustrated in Figure 1B.

#### Arousal

In the analysis examining human eating congruent stimuli and animal eating congruent stimuli, participants with misophonia found the sounds overall as more arousing (*M* = 2.42, *SE* = 0.11) than did controls (*M* = 3.18, *SE* = 0.13), Group *F*(1, 104) = 20.918, *p* < 0.001; η^2^*_*p*_* = 0.1 (lower value means higher arousal). There was no interaction between the Group and other variables.

There was an interaction of Stimuli × Presentation *F*(1, 104) = 4.342, *p* = 0.04; η^2^*_*p*_* = 0.04. While there was no difference in arousal during the human eating rating between audio and audio-video conditions (*M* = 2.7, *SE* = 0.12 vs. *M* = 2.81, *SE* = 0.12, *p* = 0.318), animal-eating sounds were assessed as less arousing in the audio-video condition (*M* = 3.05, *SE* = 0.1, *p* < 0.001) than in the audio condition (*M* = 2.64, *SE* = 0.1), *p* < 0.001. Nonetheless, there was neither a difference in arousal self-report between the stimuli in the audio condition (*p* = 0.539), nor in audio-video condition (*p* = 0.059). The data are illustrated in Figure 2A.

In the analysis examining human eating congruent stimuli and human mouth smacking incongruent stimuli, participants with misophonia also reported the sounds overall as more arousing (*M* = 2.41, *SE* = 0.11) than did controls (*M* = 3.2, *SE* = 0.13), Group *F*(1, 104) = 21.37, *p* < 0.001; η^2^*_*p*_* = 0.17. There was an interaction of Stimuli × Presentation *F*(1, 104) = 7.08, *p* < 0.009; η^2^*_*p*_* = 0.06. Pairwise comparison showed that while there was no difference between the stimuli in the audio condition (*p* = 0.35), in the audio-video condition, congruent human eating (*M* = 2.81, *SE* = 0.12) was assessed as significantly more arousing than incongruent human mouth smacking sounds (*M* = 3.09, *SE* = 0.11), *p* = 0.016.

Moreover, while there was no difference in arousal during the human eating rating between audio and audio-video conditions (*p* = 0.318), human mouth smacking sounds were assessed as less arousing in the incongruent audio-video condition (*M* = 3.09, *SE* = 0.11), than in audio condition alone (*M* = 2.61, *SE* = 0.09), *p* < 0.001. The data are illustrated in Figure 2B.

#### Dominance

In the analysis involved human eating congruent and animal eating congruent stimuli, participants in the control group reported feeling more dominant with respect to the sounds (*M* = 3.26, *SE* = 0.12) than participants in the misophonia group (*M* = 2.5, *SE* = 0.1), *F*(1, 104) = 24.119, *p* < 0.001, η^2^*_*p*_* = 0.19. There was neither an interaction between Stimuli × Presentation nor an interaction between Group status and other variables. The data are illustrated in Figure 3A.

In the analysis of human eating congruent and human mouth smacking incongruent stimuli, participants in the control group reported feeling more dominant with respect to the stimuli (*M* = 3.26, *SE* = 0.12) than participants in the misophonia group (*M* = 2.45, *SE* = 0.1), *F*(1, 104) = 25.861, *p* < 0.001, η^2^*_*p*_* = 0.2. There was no interaction between Stimuli × Presentation, but there was an interaction of Group status, Stimuli, and Presentation *F*(1, 104) = 4,32, *p* < 0.04, η^2^*_*p*_* = 0.04. Whereas in controls, there was no difference in feelings of dominance between Stimuli in the audio nor in the audio-video condition (*p* = 0.114; *p* = 0.77), participants with misophonia reported the same level of feeling dominant toward the sounds in the audio condition (*p* = 0.33), but when the stimuli were presented with videos, they felt more dominant in the case of incongruent mouth smacking sounds than during congruent human eating sounds (*M* = 2.33, *SE* = 0.13 vs. *M* = 2.8, *SE* = 0.12), *p* = 0.016. The data are illustrated in Figure 3B.

### Psychophysiological data

Although the physiological data were not distributed normally, a parametric mixed ANOVA was performed with Group (misophonia vs. control) as a between-subjects factor and Time (eight 1-s periods), Presentation (audio, audio-video), and Stimuli (human eating, animal eating) as within subjects’ factors, to test the effect of adding visual information to the actual sound’s source (congruent stimuli) on the psychophysiological reaction. Additionally, a similar mixed ANOVA was conducted with Group (misophonia vs. control) as a between-subjects factor and Time (eight 1-s periods), Presentation (audio, audio-video), and Stimuli (human eating, human mouth smacking) as within subjects’ factors, in order to examine whether presenting an actual human mouth smacking with an incongruent cue had an influence on the psychophysiological reaction.

When the sphericity assumption was not met, Greenhouse-Geisser corrected degrees of freedom and epsilon values were reported. Bonferroni *post hoc* tests with correction for multiple comparisons were conducted. These analyses were made separately for HR and SCR data.

#### Heart rate

**FIGURE 4:**
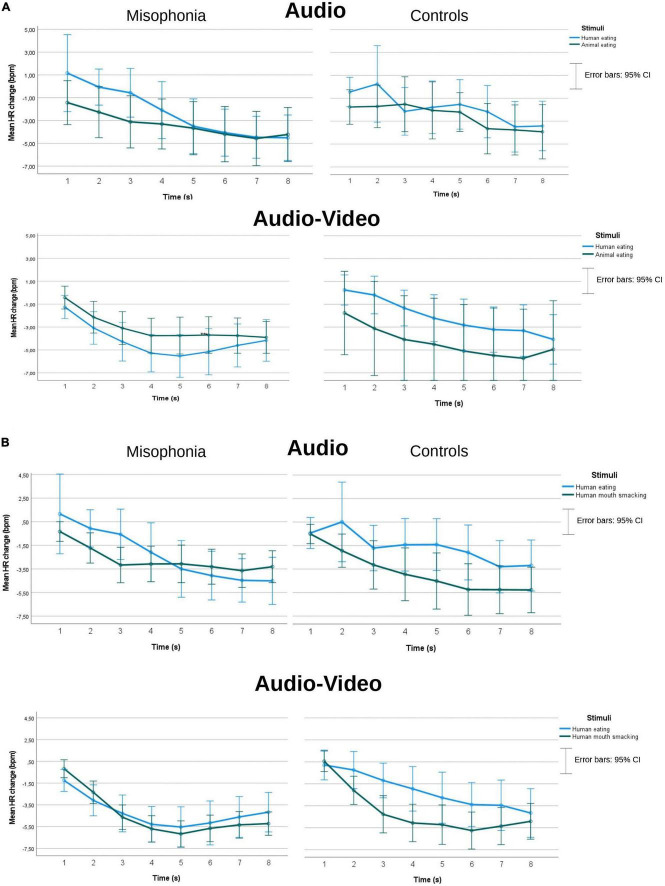
Mean HR changes (bpm) separately for misophonia and controls and two kinds of presentations (audio and audio-video), and for two separate analyses **(A,B)**.

Because the cardiac responses of 10 participants were of low quality or not recorded due to technical errors, the data gathered from 55 participants with misophonia and 42 controls were analyzed. There were no main group effects in either of the two analyses described above as (a) and (b), which means that we could not confirm the difference in the HR reaction to the stimuli between people with misophonia and controls. mean HR changes (bpm) separately for misophonia and controls, two kinds of presentations (audio and Audio-Video), and for two separate analyses (a and b) can be seen in Figure 4.

In the analysis with human eating (congruent), animal eating (congruent), and Time as within subject’s factors, only a Time effect^4^ was found *F*(3.26, 309.84) = 33.3, *p* < 0.001, η^2^ = 0.26, ε = 0.47. Pairwise comparisons indicated a deceleratory HR response to the stimuli, with HR dropping (*M* = −0.708, *SE* = 0.34) until the seventh second (*M* = −4.21, *SE* = 0.489). There was a significant difference between 2 consecutive periods: 2nd vs. 3rd second (*M* = −1.54, *SE* = 0.387 vs. *M* = −2.52, *SE* = 0.41; *p* = 0.006) and 3rd vs. 4th second (*M* = −3.12, *SE* = 0.48; *p* = 0.045).

In the analysis with human eating (congruent), human mouth smacking (incongruent), and Time as within subjects’ factors, the Time effect also showed decelatory HR response *F*(2.67, 250.54) = 49, *p* < 0.001, η^2^ = 0.34, ε = 0.38. Pairwise comparisons showed that HR was dropping (*M* = −0.905, *SE* = 0.31) until 8 s (*M* = −4.34, *SE* = 0.38). There was a significant difference between 3 consecutive periods:1st vs. 2nd second (*M* = −0.905, *SE* = 0.31 vs. *M* = −1.37, *SE* = 0.31; *p* = 0.005), 2nd vs. 3rd second (*M* = −2.870, *SE* = 0.34; *p* < 0.001), and 3rd vs. 4th second (*M* = −3.57, *SE* = 0.41; *p* = 0.009).

We also found a significant interaction of Group, Stimuli, Presentation, and Time *F*(2.95, 277.63) = 3.001, *p* = 0.032, η^2^*_*p*_* = 0.031, ε = 0.422. Pairwise comparisons showed that misophonia participants had significantly deeper HR deceleration than controls in the audio-video human eating condition in the 2nd (*M* = −3.08, *SE* = 0.714 vs. *M* = −0.242, *SE* = 0.83, *p* = 0.011), the 3rd (*M* = −4.28, *SE* = 0.78 vs. *M* = −1.19, *SE* = 0.9, *p* = 0.011), and the 4th second (*M* = −5.28, *SE* = 0.84 vs. *M* = −1.95, *SE* = 0.97, *p* = 0.011), while there was no difference between the groups in the human mouth smacking incongruent stimuli in the audio-video condition. There was no Group difference in the audio condition. A non-parametric Mann Whitney’s *U*-test confirmed the group difference in the 2nd second (*Mdn* = −2.25, *n* = 55 vs. *Mdn* = −0.75, *n* = 42), *U* = 780.50, *z* = −2.73, *p* = 0.006, and in the 3rd second (*Mdn* = −3.67, *n* = 55 vs. *Mdn* = −1.33, *n* = 42), *U* = 869.5, *z* = −2.08, *p* = 0.038. Similar analysis showed only a statistical tendency in the 4th second (*p* = 0.088).

#### Skin conductance response

As data from several participants had to be excluded due to recording errors, the results from 54 participants with misophonia and 41 controls were analyzed.

There was no Group main effect in the analysis of human eating (congruent) and animal-eating (congruent) stimuli (*p* = 0.344), nor in the analysis of human eating (congruent) and human mouth smacking (incongruent) stimuli (*p* = 0.115). In the analysis with human eating (congruent), animal eating (congruent), and Time as within subject’s factors, there was an interaction of Group, Time, and Presentation, *F*(2.66, 255.28) = 2.81; *p* = 0.046, η^2^ = 0.028, ε = 0.380, showing that misophonia participants (*M* = 0.04, *SE* = 01) had higher SCR than controls (*M* = 0.003, *SE* = 0.012; *p* = 0.023) in the 8th second in the audio condition. However, non-parametric tests, which were additionally conducted due to non-normal distribution of the data, did not confirm this difference. A Mann Whitney’s *U*-test indicated that there was no significant difference between misophonia and controls, *U* = 930.00, *z* = −1.766; *p* = 0.077.

**FIGURE 5:**
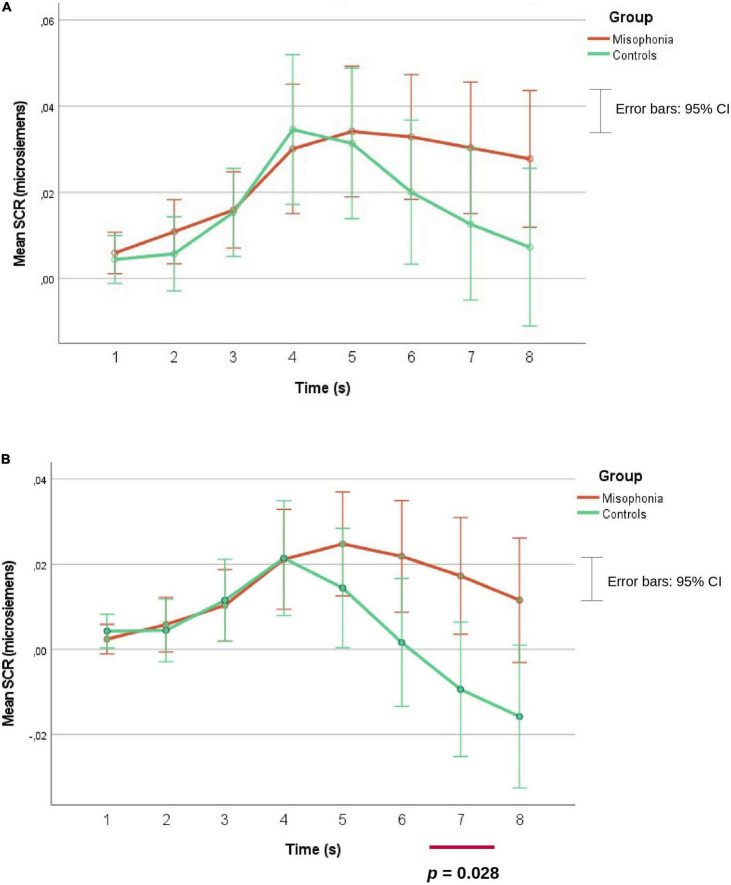
SCR means separately for misophonia and controls, and for two separate analyses **(A,B)**.

In the analysis of human eating (congruent) and human mouth smacking (incongruent) an interaction of Time and Group was found *F*(1.89, 176.02) = 4.69; *p* = 0.012, η^2^ = 0.048, ε = 0.270, indicating that participants with misophonia had significantly higher SCR than controls in the 6th (*M* = 0.022, *SE* = 0.007 vs. *M* = 0.002, *SE* = 0.008), 7th (*M* = 0.017, *SE* = 0.007 vs. *M* = −0.009, *SE* = 0.008) and 8th (*M* = 0.012, *SE* = 0.007 vs. *M* = −0.016, *SE* = 0.008) second, as can be seen in Figure 5. A Mann Whitney’s *U*-test did not confirm the difference in the 6th second (*p* = 0.162) and indicated only statistical tendency in the 8th second (*p* = 0.060) but confirmed the difference in the 7th second (*Mdn* = 0.0035, *n* = 54 vs. *Mdn* = −0.0007, *n* = 41), *U* = 815, *z* = −2.194, *p* = 0.028.

## Discussion

This study aimed to examine whether the context of the sound will affect the misophonic response. As hypothesized, the manipulations of the context of the sounds influenced the self-reported misophonic reaction. Nonetheless, among non-misophonic participants, similar effects were observed (with an exception for dominance rating). Although such a result does not support a part of our hypothesis which states that the eating sound’s context will not affect the control group, it is in line with three studies (Heller and Smith, 2022; Samermit et al., 2022; Savard et al., 2022) published after our study had begun, showing that the same orofacial or unpleasant sounds are assessed more positively when perceived or presented as something else, in participants without misophonia as well. In our study, however, controls, on average, assessed stimuli as neutral to somewhat positive in the audio-video condition. It could be assumed that a dog eating a watermelon or a pig eating from a human hand could be viewed as humorous. Participants with misophonia rated all these sounds as negative, or slightly negative (in case of animal eating and human mouth smacking sounds), even when the sounds were presented with videos.

Not surprisingly, participants with misophonia assessed all misophonic sounds as more negative and arousing, and assessed feeling less dominant with respect to sounds compared to healthy control participants without misophonia, which supports the first hypothesis and is consistent with previous studies and descriptions of misophonia (e.g., Edelstein et al., 2013; Brout et al., 2018; Swedo et al., 2022). It also confirms an adequate group assignment based on the face-to-face misophonia interview. Additionally, proper group assignment was confirmed by the significant differences in MisoQuest outcomes between the two groups.

The results of the study are consistent with the role of context of the sounds on evaluation of misophonia trigger sounds found in other studies (Frank and McKay, 2019; Edelstein et al., 2020; Natalini et al., 2020; Wiese et al., 2021; Cowan et al., 2022; Heller and Smith, 2022; Samermit et al., 2022; Savard et al., 2022). Put simply, the misophonic reaction is reduced when it is perceived as something apart from a human mouth sound. In our study, when people with misophonia (and controls as well) were not told the source of the sounds they were listening to (i.e., in the audio condition), there was no difference between human eating, human mouth smacking and animal eating sounds on the self-report valence rating. However, when exposed to the same sounds in the audio-video condition, sounds made by animals (congruent) and human smacking sounds shown as being made by human hands (incongruent) were rated as significantly less negative than human eating sounds (congruent). Moreover, while there was no difference in the valence rating between the same (congruent) human eating sound in the audio and audio-video conditions, presenting the human mouth smacking with incongruent visual stimuli significantly decreased negative affect and arousal. Similarly, in the case of animal-eating sounds, exactly the same sounds were assessed as less negative and less arousing when presented with video of congruent stimuli. Moreover, people with misophonia reported feeling more dominant toward the smacking mouth sounds with incongruent visual stimuli than toward human eating sound presented with congruent stimuli, while this effect was not observed in controls. Thus, the third and the fifth hypotheses were supported. Furthermore, in both of the groups, presenting the sounds with videos decreased reported arousal, in comparison to the audio condition, to congruent animal and incongruent human mouth smacking sounds, but not to the congruent human eating sound. This supports a part of the fifth hypothesis in this study.

Although in our study the manipulation of context involved different stimuli (e.g., audio only vs. audio-visual), the results are similar when this manipulation is carried out by text (e.g., verbally informing participants about the source of the sound; Edelstein et al., 2020). This suggests that perception of the sound’s context, rather than the specific acoustic characteristics, is a source of the evaluative differences in how people with misophonia perceive triggering stimuli.

While the self-assessment results were in line with previous studies and consistent with the misophonia reaction being affected by the context of the sound, psychophysiological data were less clear. Although parametric tests indicated several differences between participants with misophonia and controls in SCR, non-parametric test confirmed only one difference—participants with misophonia had higher SCR than controls in the 7th second in the average of audio and audio-video of human eating and mouth smacking eating stimuli. Therefore, the second hypothesis was supported only partially. The results were not as clear as in the previous studies (Edelstein et al., 2013; Kumar et al., 2017) of greater skin conductance increases among people with misophonia while listening to misophonia trigger stimuli. Furthermore, the context modification did not impact the SCR data in our study. Further studies should verify whether these findings were a result of lower statistical power of non-parametric tests, or rather in the first seconds of the trigger duration differences in SCR between people with and without misophonia are less demonstrable.

The heart rate results were also more difficult to explain and better interpreted as being related to the cognitive and attentional processing of the stimuli. In most of the analyses, no differences between the misophonia and control groups were found in the HR responses. The only difference that was found indicated more robust HR deceleration in misophonia participants than in controls during 2 s in congruent human eating sound in audio-video condition. This result, however, contradicts our hypothesis about less pronounced deceleration during human eating sounds, and may rather suggest an orienting response. The orienting response indicates attention and information intake, and is larger when stimuli are novel, interesting, or significant (Graham, 1992, 1979). These outcomes may indicate that the fight or flight response to misophonic triggers is preceded by increased attentional focus to misophonic triggers. Overfocus on triggers, and difficulties with attention shifting, was already described as a significant symptom of misophonia (Swedo et al., 2022). Nonetheless, this result was observed only in 2 s, and only in the audio-video condition, so should be treated as preliminary until replicated in further studies.

Two studies (Kumar et al., 2017; Schröder et al., 2019) have found HR increases in people with misophonia (but not healthy controls) while listening to misophonia trigger stimuli. Several differences between those studies and the current one may explain the discrepant results. First, these earlier studies employed longer stimulus presentation times (15 or 25 s) than the current study. The typical HR response to an aversive stimulus is cardiac deceleration (orienting response) during stimulus intake followed by acceleration (defensive response) in preparation for action (Bradley et al., 2001; Witvliet and Vrana, 2007), which was also observed in our data. Therefore, a longer stimulus presentation may have captured a defensive response that might have discriminated between groups or the different trigger sounds. Further, in addition to the longer presentation times, the other studies (Kumar et al., 2017; Schröder et al., 2019) repeated the stimuli multiple times, allowing for sensitization to the trigger sounds and more opportunity to observe a HR difference between groups. This study aimed to examine the immediate reactions in both groups, before it would habituate or sensitize in either of the groups. Another reason for this methodological choice was that the main goal of the study was to evaluate the effect of sounds’ source manipulation on emotional reaction. If the stimulus was repeated, there was a risk that the participants would discover the experimental manipulation, which would affect psychological assessment of the stimuli.

Another possible explanation of between-study HR differences is that stimuli were processed differently. Subtle differences in perceptual and cognitive processing can greatly affect HR response to stimuli (Vrana et al., 1986; Peasley-Miklus and Vrana, 2000). In this study, participants were given minimal instructions regarding how to process the stimuli, though the potential affective components of the stimuli were highlighted (see “Procedure” section). Other studies have not provided processing instructions, but after every trial, misophonic participants in Kumar et al. (2017) were asked to rate how annoying the sound was and how effective it was in triggering misophonic reaction, so participants were oriented toward evaluating it for a negative emotional reaction. It is recommended that future studies exploring the physiological response to misophonia stimuli publish the instructions they provide to participants about the stimuli, in order to facilitate interpretation of results and comparison across studies.

This study has several limitations that must be noted. First, the groups were not completely equivalent; people in the misophonia group were slightly older and more likely to be female compared to participants in the control condition. Second, there were no neutral, positive, or non-mouth negative stimuli to compare with the misophonia triggers. This made it difficult to find group differences or to definitively interpret the control group findings. Third, because we wanted to prevent participants from guessing the goal of the study, participants’ interpretation of the sources of the sounds were not controlled. This limitation, however, made it impossible to conclude about possible assumptions on the sound context made by the participants. Moreover, the manipulation of sound source was confounded with both sensory modality of the stimulus (audio-only and audio + visual) and with presentation order (the audio condition was always presented first). Future studies of context and interpretation of sounds on misophonia response should be designed so that equivalent stimuli can be used when awareness is manipulated, and so conditions can be adequately counterbalanced. In addition, relatively few triggers were presented, and they were presented for a short period of time because the main goal of the experiment was to evaluate immediate perception and cognitive evaluation of the stimuli. However, this had some important consequences: the methodological differences between this study and other studies make it difficult to compare the HR results. Furthermore, the fact that mouth smacking sounds were recorded without food inside the mouth, while these stimuli were supposed to be human eating sounds, possibly could not be treated and described as outright human eating sounds, which somewhat limits the interpretation of the data related to these stimuli. Moreover, due to an error, one of the human eating stimuli was presented incorrectly, so only the data from one of the human eating sound was calculated, and the average of two animal-eating stimuli and of two human smacking was calculated. A final limitation is the ecological validity of the study. The misophonia sounds were presented for only 8 s each, a much shorter duration than is typical in real life, and several participants commented that the sounds were much less unpleasant than in a real-life because they knew that it was made by an “actor.”

Despite these limitations, the study adds to the growing misophonia literature by demonstrating that the same eating sounds are assessed by misophonia sufferers as being less negative when embedded within videos of non-human eating. These contextual effects occurred quickly during sound presentations that were shorter than typically occur in real life. In future studies, the duration and maintenance of these effects should be explored. A recently developed database of potential trigger sounds paired with neutral or pleasant videos (Samermit et al., 2022) could help further studies to replicate and extend those of the present study. Additionally, our study results encourage the development of cognitive interventions for misophonia (see also Edelstein et al., 2020; Savard et al., 2022), in which interpretation and attribution of the sound is addressed. Importantly, here we only suggest that the misophonic reaction could potentially be modified by cognitive reappraisal, but we do not believe that the misophonic reaction can be removed by cognitive restructuring, or that misophonia could simply be cured with cognitive therapies.

In this study, we focused only on the source of sounds. In further studies, investigating other moderators of the misophonic response to triggering cues, such as personal experience, mental state, an attitude to specific behaviors related to the trigger sounds, etc., could extend the understanding of the context in misophonia. Empirical verification of the role of context in misophonic responses is fundamental for the understanding of the misophonia mechanism, and can contribute to developing adequate misophonia treatment.

## Data availability statement

The raw data supporting the conclusions of this article will be made available by the authors, without undue reservation.

## Ethics statement

The studies involving human participants were reviewed and approved by the Ethics Committee at the Faculty of Psychology University of Warsaw (no. 29/05/2018). The patients/participants provided their written informed consent to participate in this study.

## Author contributions

MS designed the study, led the experiment and data collection, performed the statistical analysis, and wrote the first draft of the manuscript. AR and MS processed the psychophysiological data. SV, AR, and MR modified the first draft of the manuscript and wrote sections of the manuscript. SV, WD, and MR supervised the manuscript writing. All authors contributed to manuscript revision, read, and approved the submitted version.

## Footnotes

1 Median.

2 There are no non-parametric tests for interaction, and this procedure was needed to test hypotheses in this study. Therefore, due to evidence for the adequacy and robustness of ANOVA in case of non-normally distributed data (Schmider et al., 2010; Blanca et al., 2017; George and Mallery, 2019), ANOVAs are presented despite non-normal distributions of the data. However, when the data from parametric and non-parametric tests differed, additionally, non-parametric results are presented for the specific comparisons.

3 Due to an exploratory character of the study, even when no Group interactions were found, in case of all the analyses the interactions with Stimuli and Presentations were tested, in order to check in what way the context of the sounds impacted both of the groups.

4 Due to an exploratory character of this study, in spite of no Group effects, additional interactions were tested in order to check the direction of HR changes across Time.
